# Supplementary material for: Chronic Intermittent Hypoxia Increases Cell Proliferation in Hepatocellular Carcinoma
Source: Cells. 2022 Jun 28;11(13):2051. doi: 10.3390/cells11132051 (PMC9265377; doi:10.3390/cells11132051)
Supplement: Supplementary file 1 [file cells-11-02051-s001.zip › cells-1767016-supplementary.pdf]

Supplementary Material

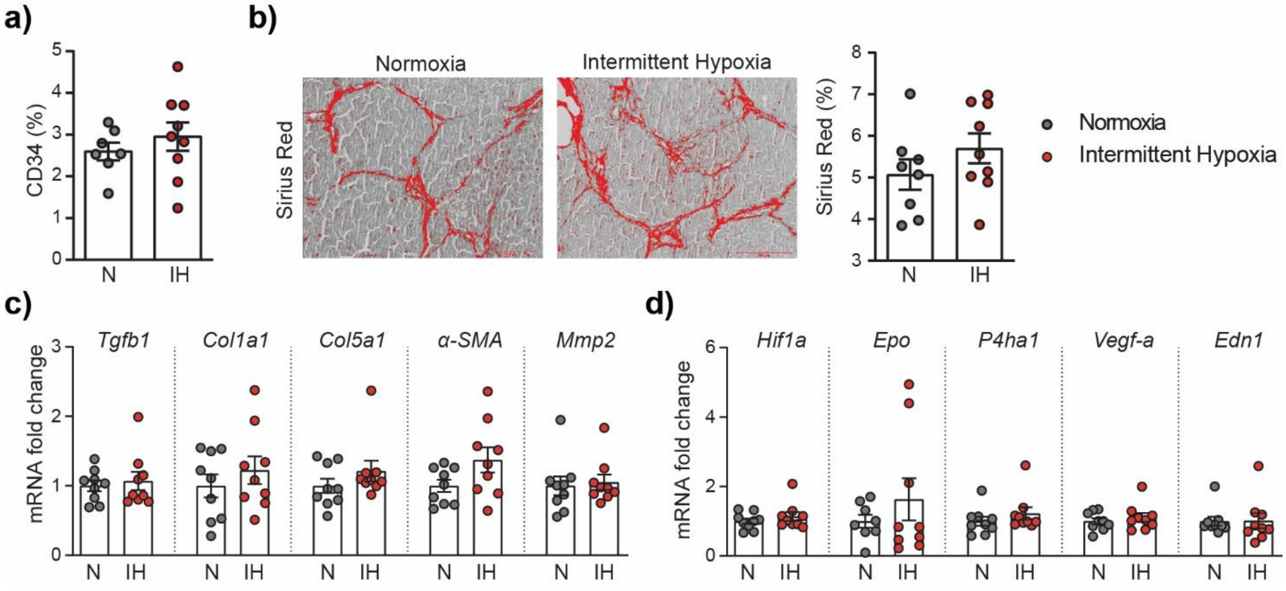

Figure S1. The impact of intermittent hypoxia on **a**) vascularization determined by CD34 staining and **b**) fibrosis status determined by Sirius red staining, **c**) qPCR analysis of fibrosis related genes and **d**) qPCR analysis of genes related to HIF-1 signaling. Each circle represents an individual animal, mean ± SE, n = 9/group. Comparison of means was performed by unpaired t test.

Table S1. Table of predesigned TaqMan probe (Thermofisher).

**Gene name**	**TaqMan probe** / **Reference**
**Ccnd1**	Rn00432359_m1
**Pbk**	Rn01534901_m1
**Mybl2**	Rn01750242_m1
**Tgfb**	Rn00565937_m1
**Col1a1**	Rn01463848_m1
**Col5a1**	Rn00593170_m1
**Acta2 (a-SMA)**	Rn01759928_g1
**Mmp2**	Rn01538170_m1
**Vegfa**	Rn01511602_m1
**Edn1**	Rn00561129_m1
**Hif1a**	Rn01472831_m1
**P4ha1**	Rn00680265_m1
**Epo**	Rn01481376_m1

Table S2: The subset of genes that contributes most to the enrichment result of Hypoxia gene set in 14+6w DEN liver versus NoDEN liver.

	Name	Rank in gene list	Rank metric score	Running enrichment score	Core enrichment
1	CA12	78	5.317	0.0233	Yes
2	MAFF	89	5.117	0.0509	Yes
3	PKP1	129	4.652	0.0737	Yes
4	AMPD3	153	4.385	0.0962	Yes
5	NDRG1	184	4.199	0.1171	Yes
6	ANXA2	226	3.910	0.1356	Yes
7	GPC3	246	3.753	0.1549	Yes
8	TES	251	3.706	0.1751	Yes
9	PGAM2	277	3.597	0.1931	Yes
10	LOX	288	3.555	0.2121	Yes
11	PDK3	456	2.917	0.2150	Yes
12	VLDLR	479	2.832	0.2290	Yes
13	FOSL2	505	2.770	0.2424	Yes
14	RRAGD	526	2.732	0.2559	Yes
15	HAS1	531	2.716	0.2707	Yes
16	ATF3	536	2.701	0.2854	Yes
17	SERPINE1	542	2.690	0.2999	Yes
18	HK1	561	2.647	0.3132	Yes
19	PFKFB3	634	2.491	0.3213	Yes
20	ISG20	690	2.371	0.3301	Yes
21	FOS	809	2.208	0.3329	Yes
22	BCL2	824	2.189	0.3440	Yes
23	STC2	834	2.174	0.3553	Yes
24	GPC1	890	2.101	0.3626	Yes
25	PLAUR	895	2.090	0.3739	Yes
26	LXN	1060	1.906	0.3715	Yes
27	PFKP	1091	1.876	0.3795	Yes
28	DDIT4	1120	1.844	0.3875	Yes
29	KDELR3	1157	1.812	0.3947	Yes
30	PHKG1	1175	1.783	0.4033	Yes
31	KLF6	1196	1.765	0.4115	Yes
32	ADORA2B	1220	1.744	0.4193	Yes
33	AKAP12	1255	1.715	0.4261	Yes
34	ADM	1270	1.705	0.4345	Yes
35	JUN	1361	1.627	0.4364	Yes
36	IGFBP3	1462	1.560	0.4371	Yes
37	PDGFB	1486	1.544	0.4438	Yes
38	F3	1491	1.539	0.4521	Yes
39	ALDOA	1553	1.498	0.4555	Yes
40	HS3ST1	1639	1.439	0.4568	Yes
41	IGFBP1	1644	1.436	0.4644	Yes
42	STC1	1684	1.407	0.4691	Yes
43	DCN	1696	1.400	0.4760	Yes
44	S100A4	1762	1.355	0.4784	Yes
45	DPYSL4	1864	1.297	0.4775	Yes
46	INHA	1878	1.292	0.4837	Yes

Table S3: The subset of genes that contributes most to the enrichment result of Hypoxia gene set (14+6w DEN Tumor versus 14+6w DEN Non Tumoral)

	Name	Rank in gene list	Rank metric score	Running enrichment score	Core enrichment
1	NDRG1	191	1.976	0.0096	Yes
2	IL6	194	1.963	0.0340	Yes
3	PGAM2	197	1.961	0.0584	Yes
4	GPC1	242	1.841	0.0779	Yes
5	AMPD3	307	1.647	0.0934	Yes
6	ANXA2	312	1.633	0.1135	Yes
7	TES	361	1.585	0.1296	Yes
8	PKP1	382	1.566	0.1476	Yes
9	PDK3	384	1.563	0.1670	Yes
10	CA12	416	1.502	0.1834	Yes
11	PHKG1	418	1.496	0.2020	Yes
12	SERPINE1	435	1.464	0.2190	Yes
13	ADM	523	1.318	0.2286	Yes
14	IGFBP3	535	1.299	0.2440	Yes
15	HAS1	560	1.267	0.2580	Yes
16	HK1	652	1.219	0.2660	Yes
17	DDIT4	685	1.181	0.2782	Yes
18	ISG20	733	1.135	0.2887	Yes
19	MAFF	734	1.135	0.3029	Yes
20	LOX	772	1.107	0.3138	Yes
21	KDELR3	773	1.106	0.3277	Yes
22	F3	861	1.048	0.3339	Yes
23	ATF3	879	1.039	0.3455	Yes
24	GPC3	914	1.023	0.3556	Yes
25	INHA	946	1.003	0.3657	Yes
26	PLAUR	1042	0.938	0.3700	Yes
27	PFKFB3	1070	0.926	0.3794	Yes
28	PGF	1092	0.912	0.3891	Yes
29	STC2	1095	0.910	0.4004	Yes
30	PFKP	1123	0.891	0.4094	Yes
31	FOSL2	1145	0.878	0.4187	Yes
32	VLDLR	1383	0.830	0.4103	Yes
33	ALDOA	1421	0.810	0.4175	Yes
34	DPYSL4	1448	0.791	0.4254	Yes
35	RRAGD	1551	0.743	0.4266	Yes

Table S4: The subset of genes that contributes to the enrichment result of Hallmark Myc Targets genesets induced by intermittent hypoxia in tumor tissue compared to tumor tissue under normoxia conditions.

HALLMARK_MYC_TARGETS_V1					HALLMARK_MYC_TARGETS_V2				
	Name	Rank in gene list	Rank metric score	Running enrichment score		Name	Rank in gene list	Rank metric score	Running enrichment score
1	POLD2	367	1.034	0.0002	1	MCM4	447	0.898	0.0111
2	LSM2	378	1.006	0.0020	2	HSPD1	556	0.807	0.0446
3	CDC20	418	0.941	0.0138	3	HSPE1	742	0.734	0.0681

4	DUT	432	0.920	0.0273	4	NOP56	844	0.677	0.0953
5	MCM4	447	0.898	0.0405	5	IMP4	940	0.631	0.1207
6	MCM6	475	0.869	0.0521	6	WDR43	980	0.613	0.1495
7	CCNA2	516	0.837	0.0622	7	PA2G4	1050	0.583	0.1744
8	PABPC4	523	0.835	0.0750	8	PLK1	1092	0.564	0.2006
9	HSPD1	556	0.807	0.0852	9	IPO4	1130	0.548	0.2262
10	SNRPA	696	0.761	0.0861	10	TMEM97	1234	0.513	0.2448
11	CDC45	705	0.755	0.0974	11	DCTPP1	1305	0.495	0.2650
12	CAD	713	0.749	0.1088	12	AIMP2	1378	0.478	0.2842
13	MCM7	727	0.740	0.1195	13	CBX3	1422	0.467	0.3051
14	HSPE1	742	0.734	0.1300	14	PPAN	1453	0.462	0.3269
15	PCNA	750	0.726	0.1410	15	BYSL	1469	0.459	0.3496
16	APEX1	780	0.707	0.1499	16	NPM1	1567	0.440	0.3648
17	HNRNPC	790	0.702	0.1603	17	FARSA	1572	0.439	0.3874
18	ERH	800	0.696	0.1707	18	NOP2	1581	0.437	0.4096
19	RANBP1	805	0.695	0.1814	19	DDX18	1626	0.427	0.4283
20	EIF2S1	808	0.693	0.1922	20	UNG	1706	0.415	0.4437
21	CCT4	826	0.685	0.2018	21	SUPV3L1	1718	0.413	0.4644
22	TUFM	834	0.683	0.2120	22	PRMT3	1808	0.395	0.4779
23	NOP56	844	0.677	0.2221	23	SLC19A1	1818	0.392	0.4976
24	PRDX3	871	0.661	0.2305	24	CDK4	1831	0.390	0.5170
25	PPM1G	894	0.653	0.2391	25	MCM5	1853	0.388	0.5356
26	RFC4	916	0.644	0.2476	26	MYC	2007	0.365	0.5424
27	EXOSC7	961	0.621	0.2539	27	MYBBP1A	2119	0.349	0.5517
28	ODC1	962	0.621	0.2638	28	PES1	2260	0.332	0.5578
29	XPO1	970	0.618	0.2731	29	TBRG4	2306	0.324	0.5711
30	MCM2	975	0.615	0.2825	30	NIP7	2343	0.318	0.5849
31	MRPS18B	1039	0.587	0.2868	31	NOLC1	2371	0.314	0.5991
32	SRSF3	1041	0.587	0.2960	32	NOP16	2526	0.296	0.6022
33	PA2G4	1050	0.583	0.3046	33	NOC4L	2554	0.292	0.6153
34	SRSF7	1066	0.578	0.3126	34	TCOF1	2598	0.288	0.6269
35	HPRT1	1074	0.574	0.3212					
36	YWHAQ	1129	0.549	0.3255					
37	DDX21	1172	0.531	0.3306					
38	EEF1B2	1177	0.529	0.3387					
39	RRM1	1183	0.528	0.3467					
40	SET	1184	0.528	0.3550					
41	CTPS1	1192	0.525	0.3628					
42	PSMA6	1200	0.522	0.3706					
43	RPS2	1210	0.520	0.3781					
44	NCBP1	1248	0.510	0.3832					
45	HSP90AB1	1254	0.508	0.3909					
46	PTGES3	1268	0.504	0.3978					
47	PHB2	1326	0.489	0.4010					
48	C1QBP	1341	0.485	0.4076					
49	RPS10	1364	0.481	0.4135					
50	RAN	1376	0.479	0.4202					
51	AIMP2	1378	0.478	0.4277					
52	CBX3	1422	0.467	0.4316					
53	EIF4A1	1460	0.460	0.4360					
54	NPM1	1567	0.440	0.4344					

55	RNPS1	1569	0.440	0.4413					
56	SNRPA1	1617	0.430	0.4443					
57	DDX18	1626	0.427	0.4505					
58	SNRPD1	1678	0.419	0.4530					
59	FBL	1692	0.416	0.4586					
60	IMPDH2	1725	0.411	0.4625					
61	RPL6	1734	0.408	0.4684					
62	DEK	1750	0.405	0.4736					
63	BUB3	1765	0.402	0.4789					
64	PWP1	1813	0.393	0.4813					
65	TRA2B	1829	0.391	0.4863					
66	CDK4	1831	0.390	0.4924					
67	RUVBL2	1846	0.389	0.4975					
68	MCM5	1853	0.388	0.5032					
69	PABPC1	1856	0.388	0.5092					
70	RPS3	1859	0.388	0.5152					
71	PSMC6	1869	0.386	0.5206					
72	NDUFAB1	1906	0.382	0.5237					
73	CYC1	1908	0.381	0.5297					
74	IFRD1	1924	0.379	0.5345					
75	SSBP1	1944	0.376	0.5390					
76	MYC	2007	0.365	0.5398					
77	SRSF1	2022	0.363	0.5444					
78	PPIA	2035	0.361	0.5492					
79	EIF3D	2039	0.360	0.5547					
80	VDAC3	2045	0.360	0.5600					
81	MRPL9	2133	0.347	0.5585					
82	RPLP0	2164	0.343	0.5615					
83	CCT7	2179	0.341	0.5658					
84	EIF4G2	2192	0.340	0.5702					
85	RPL14	2214	0.338	0.5739					
86	GLO1	2218	0.337	0.5790					
87	PSMA1	2325	0.321	0.5755					
88	NOLC1	2371	0.314	0.5769					
89	RPL18	2404	0.310	0.5792					
90	SLC25A3	2405	0.310	0.5842					
91	EIF3B	2411	0.309	0.5887					
92	FAM120A	2464	0.303	0.5893					
93	VDAC1	2502	0.299	0.5910					
94	NOP16	2526	0.296	0.5939					
95	CCT3	2529	0.295	0.5984					
96	CLNS1A	2530	0.295	0.6031					
97	TRIM28	2561	0.292	0.6053					
98	KPNA2	2631	0.283	0.6043					
99	SRSF2	2677	0.278	0.6050					
100	TCP1	2685	0.277	0.6089					
101	CCT2	2781	0.266	0.6054					
102	CDK2	2782	0.266	0.6097					
103	PSMB2	2840	0.260	0.6092					
104	SRPK1	2875	0.257	0.6105					
105	PGK1	2898	0.254	0.6128					
106	ABCE1	2923	0.251	0.6148					

107	GOT2	2940	0.250	0.6175					
108	HDAC2	3024	0.242	0.6146					
109	HNRNPA1	3026	0.242	0.6184					
110	USP1	3101	0.236	0.6162					
111	YWHAE	3125	0.234	0.6180					
112	COX5A	3139	0.232	0.6207					
113	NME1	3151	0.231	0.6235					
114	SF3B3	3208	0.224	0.6225					
115	CCT5	3236	0.220	0.6238					
116	HDGF	3238	0.220	0.6272					
117	DHX15	3247	0.219	0.6301					
118	CANX	3258	0.218	0.6327					
119	PSMA2	3283	0.216	0.6342					
120	EIF4H	3322	0.212	0.6345					
121	SMARCC1	3373	0.208	0.6338					
122	KPNB1	3380	0.207	0.6366					
123	STARD7	3449	0.201	0.6343					
124	PSMD1	3532	0.193	0.6307					
125	HDDC2	3563	0.190	0.6313					
126	SSB	3580	0.189	0.6330					
127	RPS5	3597	0.187	0.6347					
128	PSMD14	3616	0.185	0.6362					
129	PRDX4	3651	0.182	0.6364					
130	G3BP1	3672	0.180	0.6376					
